# Acute Supplementation with High Dose Vitamin D3 Increases Serum Anti-Müllerian Hormone in Young Women

**DOI:** 10.3390/nu9070719

**Published:** 2017-07-08

**Authors:** Nicola A. Dennis, Lisa A. Houghton, Michael W. Pankhurst, Michelle J. Harper, Ian S. McLennan

**Affiliations:** 1 Department of Anatomy, School of Biomedical Sciences, University of Otago, Dunedin 9054, New Zealand; NDennis@abacusbio.co.nz (N.A.D.); ian.mclennan@otago.ac.nz (I.S.M.); 2 Department of Human Nutrition, Division of Sciences, University of Otago, Dunedin 9054, New Zealand; lisa.houghton@otago.ac.nz (L.A.H.); michelle.harper@otago.ac.nz (M.J.H.)

**Keywords:** 25(OH)D, AMH, ovary, female reproduction

## Abstract

Anti-Müllerian hormone (AMH) is a paracrine regulator of ovarian follicles. Vitamin D (Vit D) regulates AMH production in vitro, but its role as a regulator of ovarian AMH production is contentious. If Vit D influences ovarian AMH production, then an acute rise in Vit D level should lead to an acute rise in circulating AMH levels. This hypothesis was tested with a randomized double-blind design, with 18–25-year-old women recruited from the community. The study was conducted in early spring, when the marker of Vit D level (25-hydroxyvitamin D, 25(OH)D) tends to be at its nadir. The women consumed either an oral dose of 50,000 IU of Vit D3 (*n* = 27) or placebo (*n* = 22). The initial 25(OH)D ± SD value was 53.6 ± 23.3 nmol/L, with 42 of the 49 women having a value below 75 nmol/L, consistent with seasonal nadir. All women receiving Vit D3 treatment exhibited a robust increase in serum 25(OH)D within 1 day (15.8 ± 1.1 nmol/L (*n* = 27), *p* < 0.0001), with the increase sustained over the study week. Circulating levels of AMH in the women receiving Vit D3 progressively rose during the following week, with a mean increase of 12.9 ± 3.7% (*n* = 24, *p* = 0.001). The study supports the hypothesis that Vit D’s positive effects on the fertility of woman may involve the regulation of ovarian AMH levels.

## 1. Introduction

Vitamin D (Vit D) deficiency has been linked to reduced female fertility [1]. The underlying mechanism(s) may be complex, with diminished levels of ovarian anti-Müllerian hormone (AMH) being a putative component [2]. AMH is a paracrine regulator of the gonads that is also released into the circulation [3]. High levels of circulating AMH are unique to developing males [4], and contribute to male sexual differentiation [3,5]. After puberty, young men and women have similar low levels of circulating AMH [4], the physiological relevance of which is currently unclear. In women, AMH is synthesized by ovarian granulosa cells, with the levels of follicular AMH varying with the stage of follicular development [6]. AMH modulates the response of follicles to follicle-stimulating hormone (FSH) [7], with AMH deficiency leading to accelerated depletion of ovarian follicles [8]. Vit D regulates AMH levels in vitro, both directly through the AMH promoter [9] and indirectly by regulating the number of granulosa cells and AMH signaling in cultures of ovarian follicles [10]. In contrast to the consistency of the in vitro data, the evidence of a link between Vit D and AMH in women is contentious.

When AMH is acting in a paracrine mode, the biologically relevant concentration is the level of AMH within and adjacent to the sites of AMH synthesis. The measurement of AMH levels in ovarian follicles requires the removal of an ovary, necessitating the use of serum AMH. The biology of AMH needs to be taken into account when doing this, as the absolute levels of serum AMH relate to the putative hormonal functions of AMH, which are only loosely coupled to the paracrine functions of AMH. In explanation, over 80% of the inter-person variation in circulating AMH levels relates to the number of AMH-producing follicles. The number of ovarian follicles diminishes with age, reaching zero at menopause: the number of follicles that a woman has on a given day is therefore the product of historic rather than current regulation. Current vitamin D status—as measured by 25-hydroxyvitamin D (25(OH)D) levels—should strongly associate with the levels of AMH within ovarian follicles, but would be expected to only minimally associate with absolute serum AMH values, as the amount of AMH produced per follicle is only a minor determinant of circulating AMH levels. Consistent with this, cross-sectional studies have typically not detected a significant association between current 25(OH)D and current AMH values [11,12,13]. This does not disprove the hypothesis that Vit D regulates AMH production, as current serum AMH will predominantly reflect historic Vit D status, with the small influence of current 25(OH)D only being detectable in very large cross-sectional studies (see [14] for a more extensive discussion of this issue).

The number of ovarian follicles is practically constant during the first week of a menstrual period. Consequently, dynamic regulation of AMH production can be studied by determining whether acute variation in a putative regulator of AMH leads to an acute change in serum AMH. When the data is normalized to each woman’s AMH levels at the start of the study, then the resulting data measures the dynamic regulation of AMH production (AMH per follicle) with little or no influence of follicle number. We have therefore examined whether a single large oral dose of cholecalciferol (vitamin D_3_, Vit D3) taken at the start of the menstrual cycle leads to an acute increase in women’s circulating levels of AMH during the following 7 days.

## 2. Materials and Methods 

### 2.1. Study Participants

**Figure 1:**
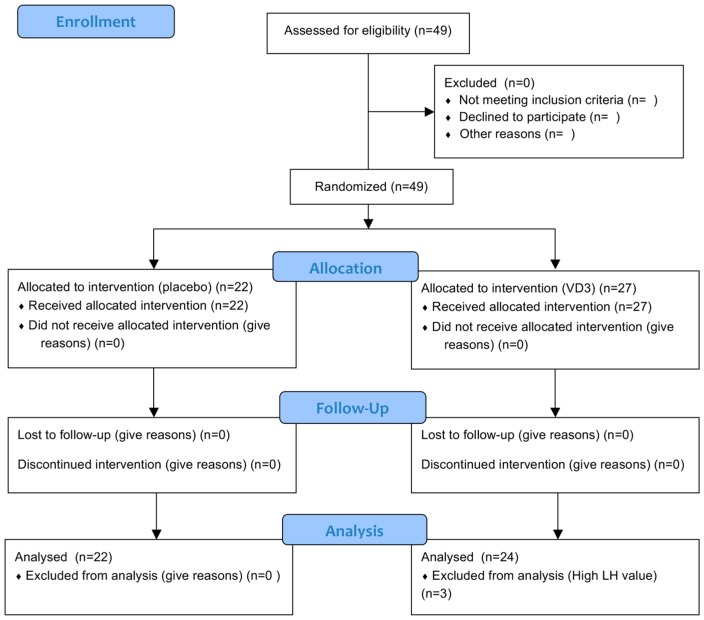
CONSORT flow diagram.

The study was conducted in early spring (20 August to 10 September) in Dunedin New Zealand (latitude: 45.9 degrees South) to ensure that 25(OH)D levels were close to the nadir in the study population. All participants were younger women recruited by advertisements in the local community. The eligibility criteria were regular menstrual cycles, not pregnant, had not breastfed within the previous two months, not taken Vit D supplements, not travelled to the Northern hemisphere, nor used sun-beds. Women were excluded if they had been diagnosed as having any history of endocrine or reproductive diseases, including polycystic ovary syndrome (PCOS). Information was recorded about current medication. Luteinizing hormone (LH) was used post-hoc to verify the self-reporting of the stage of the menstrual cycle, and to exclude women with atypically short follicular phases of their ovarian cycle: three women were excluded, as their LH value on day 7 was greater than 20 IU/L, indicating that they had progressed beyond the follicular phase of the ovarian cycle, when AMH levels begin to vary [15]. Forty-nine women were recruited, three of whom were excluded, as summarized in the CONSORT flow diagram (Figure 1).

### 2.2. Vitamin D Administration

Women consumed either a single capsule containing a dose of 50,000 IU of Vit D3 or an identical placebo (Optimus Healthcare Limited, Auckland, New Zealand) during the week after the onset of their menstrual bleeding. A permuted block randomization with a block size of ten was used to assign women to the treatment or control group. Study capsules were coded by a third party, and all participants and investigators remained blinded to the treatment groups until all statistical analyses were completed. A research nurse randomized the participants, provided each participant with a capsule containing either placebo or Vit D3, and observed consumption of the capsule. Height and weight were taken using standardized techniques, with participants wearing light clothes and no shoes. Height was measured to the nearest 0.1 cm using a calibrated self-made stadiometer, and weight was measured to the nearest 0.1 kg using a calibrated platform digital scale (Seca).

### 2.3. Blood Sampling and Assays 

Non-fasting venous blood samples were collected in vacutainers (Becton Dickinson Life Sciences, Franklin Lakes, NJ, USA) by the research nurse immediately prior to treatment, and 1, 3, and 7 days later. The samples were clotted at room temperature, centrifuged, and the resulting serum divided into aliquots to be stored at −80 °C. AMH levels were determined using the AMH Gen II enzyme-linked immunosorbent assay (Beckman Coulter, Brea, CA, USA, A79765), which measures total AMH (proAMH and AMH_N,C_ combined) [16]. The assay buffer was added to the serum before addition to the ELISA plate, in accordance with field safety notice FSN-20434-3, June 2013. Assays were conducted in duplicate, with quadratic equations used to calculate sample values from the standard curves, according to the manufacturer’s instructions. The levels of all samples were above the limit of detection of the ELISAs, with the intra-assay coefficient of variation (CV) being less than 6%. The samples were analyzed as a single batch to avoid inter-assay variation. Vitamin D levels as total 25(OH)D (25-hydroxyvitamin D_3_ plus 25-hydroxyvitamin D_2_) were measured by isotope-dilution liquid chromatography-tandem mass spectrometry based on the method of Maunsell et al. [17]. Pooled serum samples and external quality control (UTAK Laboratories, Inc., Valencia, CA, USA) were used to check the precision and accuracy. The pooled serum between-assay CV was 3.1%. Values for the low and medium controls were within the verified ranges for the both the 25(OH)D3 low value of 29.9 nmol/L (mean 28.8 (SD 1.5) nmol/L; CV 5.3%) and medium value of 79.9 nmol/L (mean 80.0 (SD 2.6) nmol/L; CV 3.3%) and the 25(OH)D2 low value of 26.6 nmol/L (mean 23.3 (SD 1.6) nmol/L; CV 6.7%) and medium value of 77.5 nmol/L (mean 69.6 (SD 3.8) nmol/L; CV 5.5%. LH levels were measured using the Elecsys LH assay and cobas e602 automated assay system (Roche) at Southern Community Laboratories, Dunedin, New Zealand.

### 2.4. Statistical Analysis

The data were normalized to each woman’s day 0 AMH and day 0 25(OH)D values, for the reasons outlined in the Introduction. Means and standard deviations cannot be directly calculated from arithmetical ratios (e.g., [day 1 AMH]/[day 0 AMH]), as decreases are represented on a scale of 0 to 1 and increases are represented on a scale from 0 to infinity. The ratios were therefore calculated using natural logs (e.g., ln([AMH day 1]) − ln([AMH day 0])), which produces a symmetrical distribution without bias to either increases or decreases. All statistical analyses were undertaken using the log ratios. Once means and standard deviations were calculated, the results were reconverted to arithmetic ratios (percentage change) using antilog (e.g., Exp(mean (In([AMH day 1]) − In([AMH day 0]))), as this provides a more natural indication of the magnitude of the observed effects.

**Figure 2:**
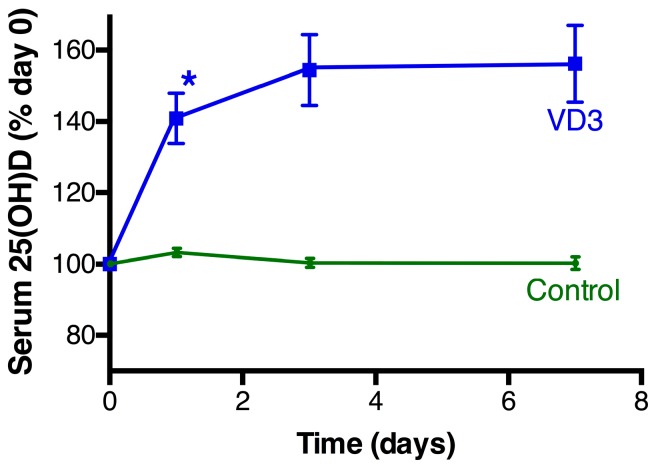
25(OH)D levels in the serum. The women either took a placebo (*n* = 22, green circles) or a tablet containing a 50,000 IU of Vit D3 (*n* = 27, blue squares) on day 0. The data is mean ± the standard error of the mean. * Significant different to control women (Paired *t*-test, *p* < 0.0001).

**Figure 3:**
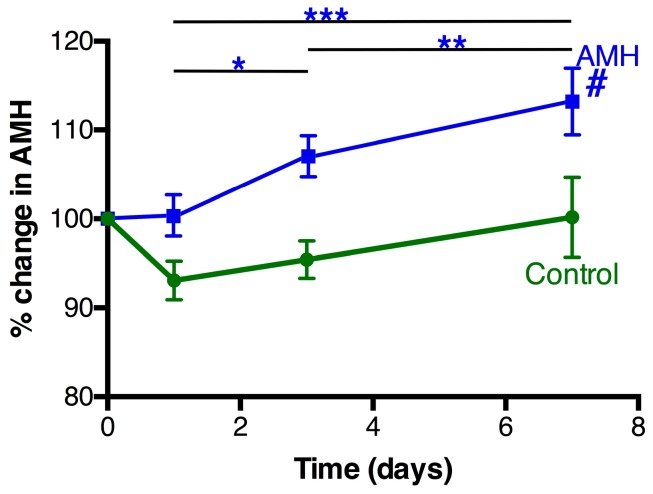
Serum AMH levels in the days following treatment. The women took either a placebo (*n* = 22, green circles) or a tablet containing a 50,000 IU of Vit D3 (*n* = 27, blue squares) on day 0 and the serum levels of AMH measured at the indicated time points. # There was a significant change in AMH level over time in the Vit D3 group (*p* = 0.001), but not in the control group. Pair-wise post-hoc analysis within the Vit D3 group (* *p* = 0.054; ** *p* = 0.033; *** *p* = 0.006).

The changes in 25(OH)D and AMH levels over time (Figure 3) were analysed using repeated-measures general linear model, with Huynh–Feldt correction. When a significant change was detected, in group pair-wise comparisons of the time points were made using Bonferroni’s correction for multiple tests. The experiment is a test of whether a biological mechanism exists (Vit D3 regulation of ovarian AMH production) which should be universal. The repeated analysis tests whether the control and Vit D3 groups are different on average, but does test whether any effect of Vit D3 on AMH occurs in most women, or only in a sub-population. AMH levels in women exhibit minor day-to-day fluctuations during the first week of the menstrual period, but with no discernible pattern [15]. In the absence of an external influence, the women’s day 7 AMH values will typically be either slightly higher or slightly lower than their day 0 value. In the control group, women exhibiting slight increases and slight decreases should be equally common, whereas the vast majority of women receiving Vit D3 should exhibit an increase, although the magnitude of the increase will vary between women. Chi-Squared tests were used to test whether women exhibiting rises and falls in their AMH levels occurred with equal frequency in both the control and Vit D3 groups. Independent-sample T-tests were used to verify that there was no difference in the characteristics of the women assigned to the control and Vit D3–treated groups (Table 1), with Levene’s test used to determine whether equal variances were assumed. Independent-sample *t*-tests were also used to test whether the women taking oral contraceptives were different to those with uncontrolled ovarian cycles. Paired *t*-tests were used to verify that the 25(OH)D levels of the women in the Vit D3 rose rapidly, as expected (Figure 2). SPSS was used for the statistical calculations (IBM Corporation, Armonk, NY, USA).

The study was approved by the University of Otago Human Ethics Committee (12/156), as a pilot study of 50 women, with the option to request expansion of the study if the observed power was insufficient. All participants provided their informed consent in writing before inclusion in the study. The investigations were carried out following the rules of the Declaration of Helsinki.

## 3. Results

### 3.1. Participant Baseline Characteristics

The women in the Vit D3-treatment and placebo groups were aged between 19 to 25 years old, and were similar in all respects (Table 1). The initial 25(OH)D levels in the cohort spanned 10–115 nmol/L, with 40 of the 46 women having values below 75 nmol/L, consistent with the seasonal decline in Vit D3 levels (Table 1). The initial AMH levels in the cohort were also diverse (13–140 pM) (Table 1), in agreement with large age-specific reference ranges [18]. The women reported that their health was good or excellent, with no known ovarian conditions. Five of the 46 women were receiving treatment for asthma (Seretide or Flixotide) (Table 1)—their data were no different from the other women.

### 3.2. Vitamin D Changes Following Treatment

Serum 25(OH)D levels in the women in the control group exhibited minor day-to-day fluctuation (range between day 0 and day 1; −6.5 to 5.6 nmol/L) with no change on average (Figure 2). All women who consumed the oral dose of Vit D3 exhibited an overt rise in their serum 25(OH)D levels at the 24-h time point (Vit D3 group: 15.8 ± 1.1 nmol/L (*n* = 24); control: 1.2 ± 0.7 nmol/L (*n* = 22), *p* < 0.0001), with the elevated level of 25(OH)D being sustained to the 7-day endpoint of the experiment (Figure 1). Some of the women’s 25(OH)D levels were still suboptimal after Vit D treatment, with the level of 25(OH)D in the Vit D3 group as a whole (Figure S1) mirroring the summer pattern for the local community [19]. No adverse effects of Vit D3 or the placebo were reported by the participants.

### 3.3. AMH Changes Following Treatment

When the women’s initial levels of AMH were analyzed as a single group (*n* = 46), there was no significant correlation with their initial serum 25(OH)D levels (*R* = 0.20), as expected (Figure S2). The mean AMH levels in the women receiving the placebo decreased slightly during the first day of the study, but there was no statistically significant change in the control group during the study period (Figure 3). In contrast, the women receiving the Vit D3 treatment exhibited a progressive rise in their level of serum AMH (*F* = 9.7, *p* = 0.001) (Figure 3). The mean change in AMH levels at 7 days was 12.9% ± 3.7% with a range − 13% to 68%: 21/24 women exhibited an increase at day 7 (significantly different to equal increase/decreases, *p* = 0.001), whereas the control group had similar numbers of women with small rises (10/22) and small declines (12/22). The mean rise in serum AMH in pmol/L in the Vit D group after 7 days was 3.3 ± 1.2 (*n* = 24, *p* = 0.010) (range − 11.5 to 17.3). The women with lower initial AMH values tended to exhibit the greater percentage rises in AMH during the 7 days, in both the placebo and Vit D groups (*R* = 0.41, *p* = 0.005).

### 3.4. Additional Factors

There was little or no difference between the women taking a hormonal contraceptive and those with natural cycles. The day 0 AMH levels were not different (contraceptive use 35.5 ± 6.6 (*n* = 21) vs. 36.1 ± 57 (*n* = 25) pM), and both groups of women exhibited a statistically significant rise in AMH following Vit D treatment (% rise at Day 7: contraceptive use 18.1 ± 7.2, *n* = 10, *p* = 0.019 vs. 9.3 ± 4.0, *n* = 14, *p* = 0.029). The magnitude of the rises in 25(OH)D (7 days: contraceptive use 20.9 ± 2.7 vs. 21.4 ± 2.4 nmol/L, *p* = 0.89) and AMH (*p* = 0.28) caused by Vit D3 treatment were not significantly different between the groups. AMH levels in the women receiving the placebo were also independent of the use of an oral contraceptive (% rise at Day 7: contraceptive use 0.3 ± 6.1, *n* = 11, *p* = 0.96 vs. 0.1 ± 7.1, *n* = 11, *p* = 0.99).

## 4. Discussion

The administration of a single oral dose of Vit D3 produced an acute change in the serum levels of 25(OH)D of young women, followed by a progressive rise in their serum AMH levels. The Vit D3 treatment moved the distribution from the winter to the summer pattern of 25(OH)D for the local population [19]. This observation supports the theory that Vit D status influences the production of AMH on a per-follicle basis, via a direct effect on the AMH gene promoter [9] and/or by increasing granulosa cell number [10]. 

AMH modulates primordial follicle activation, which is a determinant of the number of developing follicles later in life [8]. On this basis, Vit D regulation of AMH would be expected to influence the rate of ovarian reserve depletion. However, Vit D is a complex regulator with broad effects throughout the body, some of which potentially impact follicle development. Studies are therefore needed to determine if the effects of chronic reversal of Vit D deficiency affects either the quality of oocytes and/or the rate of depletion of ovarian reserve.

The Vit D3 treatment acutely changed the distribution of 25(OH)D values from a winter to a summer pattern for the local population: the average change in serum AMH (13%) that resulted was similar in magnitude to that which occurs during the middle and luteal phases of the ovarian cycle [15], with this variation being a minor contributor to the overall variation between young women. The frequency of Vit D deficiency in populations varies depending on latitude, skin colour, and culture, and the relative importance of Vit D deficiency would be expected to vary from location to location. The current study was highly powered, with the observed power (alpha 0.05) in the repeated-measures analysis being 0.94. The power arises because the study examines the response of individual women to an induced change in Vit D status, and therefore involves a direct test of whether a physiological mechanism exists. The size of the study would need to be increased by orders of magnitude to detect the mechanism by cross-sectional correlation of serum AMH and 25(OH)D levels. The women in the study all had similar ovarian status, which contributes to the observed power and also adds clarity to the result, as it does not tacitly assume that the Vit D regulation of the ovary is invariant (see below).

Clinical decisions based on serum AMH levels predominantly relate to women with limited ovarian reserve and low AMH levels. In the healthy young women in this study, the day-to-day variation in AMH levels and the proportional increase in serum AMH after Vit D treatment tended to be greater in women with AMH levels in the lower end of the range. It is currently unclear whether a similar situation will occur in women with lower ovarian reserve, as the regulation of cell behavior is dependent on the environment of the cells. Consequently, the ovarian biologies of young and older women are not necessarily identical in all aspects. Clinical trials are therefore needed to determine whether reversal of Vit D deficiency produces clinically-relevant changes in AMH levels in women with low ovarian reserve. The methodology used in this study identifies how individuals and groups respond to changes in Vit D treatment. This is advantageous compared to regression analysis of population variation, as it has the potential to reveal sub-populations of women for whom Vit D treatment has differential benefit.

## 5. Conclusions

This study reports that an acute rise in Vit D status in women leads to changes in circulating AMH that are consistent with animal and human in vitro studies. The study defines the normal physiology of young women: clinical studies are needed to determine the relevance of Vit D regulation of AMH production to ovarian pathologies and to the fertility of women with depleted ovarian reserve. 

## Figures and Tables

**Table 1:** Participant characteristics.

Group	Control	Vit D3
N	22	24
Age (years)	21.7 ± 1.4 (19.4–25.2)	21.7 ± 1.1 (19.6–24.5)
Oral contraceptive *	10	11
Asthma medication #	3	2
BMI (kg/m^2^)	22.1 ± 2.9 (17.3–29.2)	23.1 ± 2.4 (17.3–26.9)
Initial AMH (pM)	39.9 ± 30.0 (9.7–139.9)	32.0 ± 28.0 (7.7–131.6)
Initial 25(OH)D (nmol/L)	54.1± 25.9 16.1–115	51.6 ± 22.1 (9.7–109)

The data are the mean ± the standard deviation, with the range indicated in parenthesis. There is no significant difference between control and vitamin D_3_ (Vit D3)-treatment groups for any of the parameters. The control group received a placebo, and the Vit D3 group 50,000 IU of Vit D3. 25(OH)D: 25-hydroxyvitamin D; AMH: anti-Müllerian hormone; BMI: body mass index. * The number of women taking either Norimin or Ginet 84 for oral contraception. # The number of women taking Seritide or Flixotide for asthma.

## Supplementary Materials

The following are available online at www.mdpi.com/2072-6643/9/7/719/s1, Figure S1: 25(OH)D levels before and after Vit D3 treatment, Figure S2: Relationship between the women’s initial 25(OH)D and AMH levels.
