# Supplementary material for: Acute Supplementation with High Dose Vitamin D3 Increases Serum Anti-Müllerian Hormone in Young Women
Source: Nutrients. 2017 Jul 8;9(7):719. doi: 10.3390/nu9070719 (PMC5537834; doi:10.3390/nu9070719)
Supplement: Supplementary file 1 [file nutrients-09-00719-s001.zip › Dennis et al Supplementary Figures.docx]

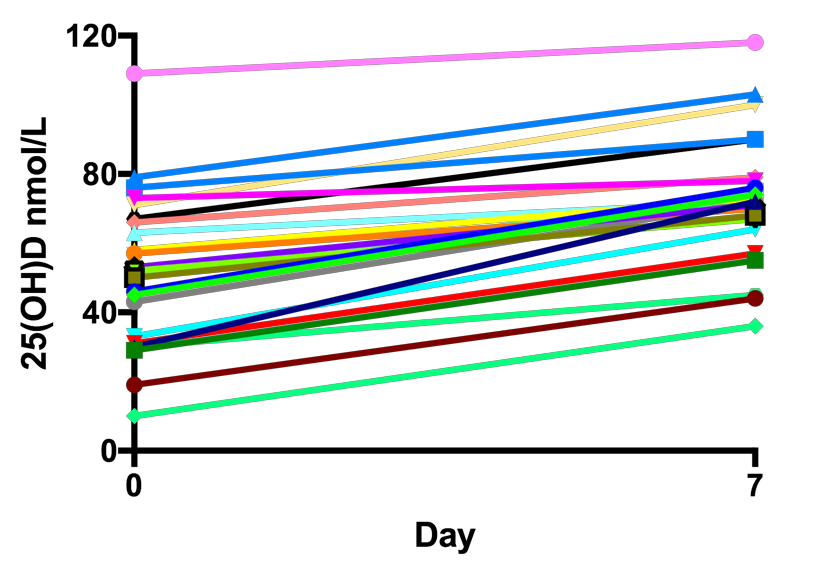


Figure S1. 25(OH)D levels before and after VD3 treatment


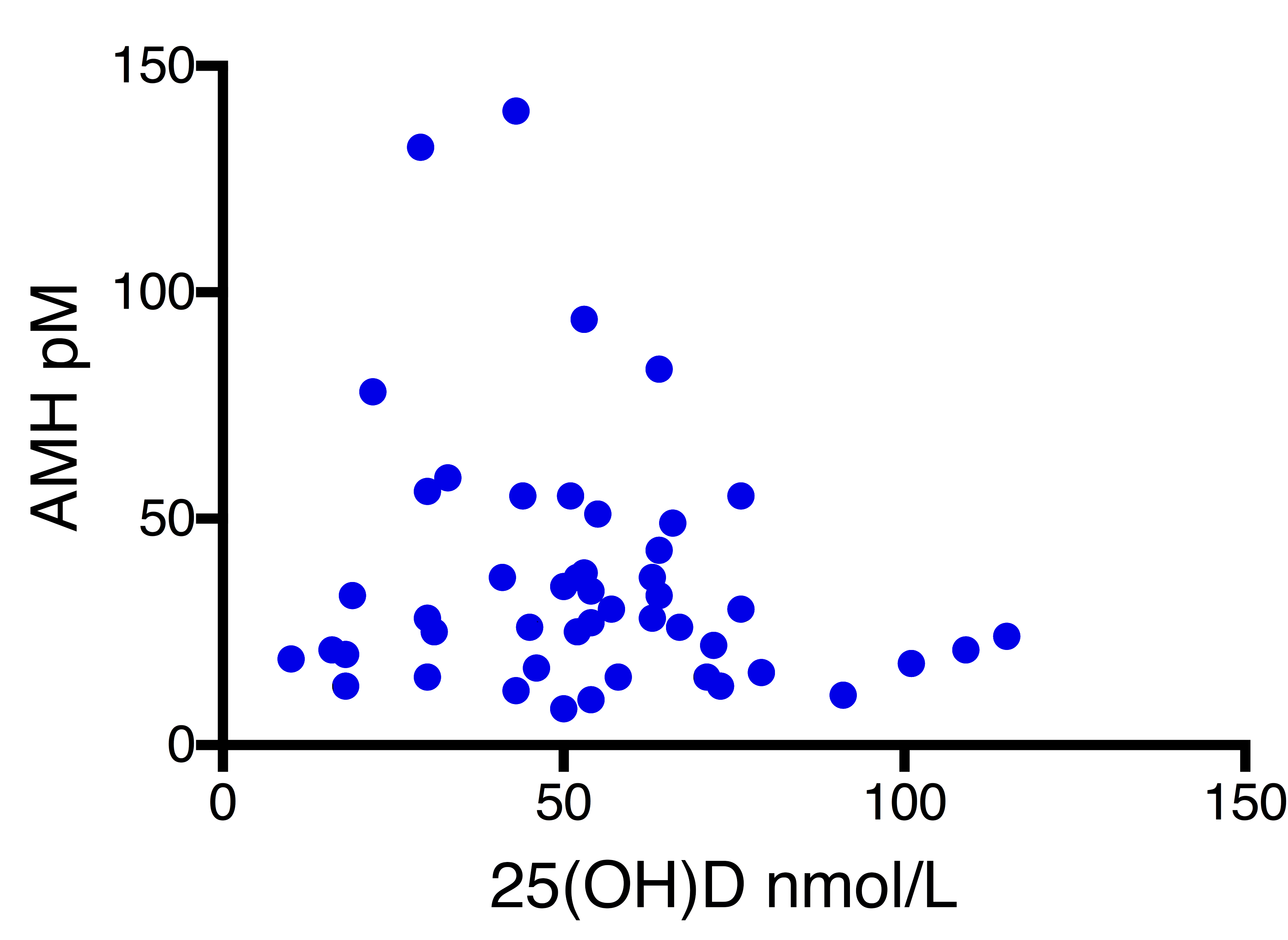


Figure S2. Relationship between the women’s initial 25(OH)D and AMH levels.
